# Preparation, Performances, and Mechanisms of Microbial Flocculants for Wastewater Treatment

**DOI:** 10.3390/ijerph17041360

**Published:** 2020-02-20

**Authors:** Huiru Li, Shaohua Wu, Cheng Du, Yuanyuan Zhong, Chunping Yang

**Affiliations:** 1 College of Environmental Science and Engineering, Hunan University and Key Laboratory of Environmental Biology and Pollution Control (Hunan University), Ministry of Education, Changsha 410082, China; lihuiru@hnu.edu.cn (H.L.); wushaohua@hnu.edu.cn (S.W.); 2 Guangdong Provincial Key Laboratory of Petrochemical Pollution Processes and Control, School of Environmental Science and Engineering, Guangdong University of Petrochemical Technology, Maoming 525000, China; ducheng@gdupt.edu.cn (C.D.); zhongyy@gdupt.edu.cn (Y.Z.); 3 Hunan Provincial Environmental Protection Engineering Center for Organic Pollution Control of Urban Water and Wastewater, Changsha 410001, China

**Keywords:** bioflocculant, bioflocculation, wastewater, water treatment

## Abstract

In recent years, close attention has been paid to microbial flocculants because of their advantages, including safety to humans, environmental friendliness, and acceptable removal performances. In this review, the preparation methods of microbial flocculants were first reviewed. Then, the performances of bioflocculants in the removal of suspended solids, heavy metals, and other organic pollutants from various types of wastewater were described and commented, and the removal mechanisms, including adsorption bridging, charge neutralization, chemical reactions, and charge neutrality, were also discussed. The future research needs on microbial flocculants were also proposed. This review would lead to a better understanding of current status, challenges, and corresponding strategies on microbial flocculants and bioflocculation in wastewater treatment.

## 1. Introduction

Advances in science and technology have increased productivity. However, every coin has two sides. When we enjoy the convenience they bring to the development of all walks of life, we must also pay attention to the environmental damage and the threat to the health of living organisms during the production process. Bioremediation is the use of living organisms, or part of it, or their metabolites, for the recovery or cleaning up of a contaminated medium such as soil [1,2], sediment [3,4], or liquid/water [5,6]. With the growth of population and the development of science and technology, more water is consumed in daily life and production, and more wastewater is generated. In recent years, both from the perspective of production requirements and environmental requirements, wastewater treatment has been an important part that cannot be ignored [7]. One of the main difficulties in wastewater treatment is that particles dispersed in water cannot be effectively separated due to their solidification stability and sedimentation stability [8]. In order to destroy its stability, some means to make the particles in the water settle down through flocculation and sedimentation is needed. The flocculants mainly include inorganic flocculants, organic polymer flocculants, and microbial flocculants (MBF) [9]. Table 1 shows the advantages and limitations of three types of flocculants. Inorganic flocculants can be divided into two systems of iron and aluminum, which have a good treatment effect on suspended solids and colloidal particles; organic polymer flocculants are mainly polyacrylamide and their derivatives, which have good treatment effects on suspended solids and coarse particles. MBF are divided into extracellular metabolic flocculants, intracellular extractive flocculants, and bacterial flocculants [10]. The scope of application is relatively wide, and some microbial flocculants have the effect of selectively treating wastewater.

For a long time, inorganic flocculants and organic flocculants are mainly used in wastewater treatment both at home and abroad [11]. However, the sludge produced by treating the wastewater with aluminum salt flocculant is often used as a fertilizer in agriculture, resulting in high aluminum content in the soil, causing soil pollution and residue in plants. More seriously, free aluminum ions enter the groundwater, rivers, and lakes through diffusion, infiltration, deposition, and migration, causing water pollution and endangering human health. Iron flocculant itself has strong corrosiveness, which will accelerate the aging of equipment and increase the cost of water treatment [7]. Organic polymer flocculant has the characteristics of less dosage, fast flocculation, but does not produce easily biodegradable residues [12]. Polyacrylamide is the most commonly used in the synthetic organic flocculants [13]. Although the polyacrylamide itself has no toxicity, its difficult degradation easily cause secondary pollution, and the residue of polymer monomer acrylamide is also a very worrying problem, as it not only has a strong neurotoxicity, but also a strong carcinogen [14]. Compared with them, microbial flocculant, which mainly refers to the microorganisms and their metabolites, has the advantages of being effective, low-cost, and a security extensive source [15,16].

Microbial flocculant is a kind of nontoxic biopolymer compound formed by a microorganism or its metabolites, major include glycoprotein, polysaccharide, protein, cellulose, DNA, as well as the flocculation activity of the bacteria [17]. Microbial flocculants are biological macromolecules, and their flocculation activity is affected by the length of the molecular chain. Generally, the longer the molecular chain, the better the flocculation effect. In addition, the molecular structure is also one of the factors affecting the flocculation effect. Due to the influence of space, the flocculation effect of the flocculant molecule with a linear structure is better than that of a branched structure or a crosslinked structure. Microbial flocculants make the suspended particles in the water settle through an adsorption bridging role. The molecular structure of the flocculants contains many hydrophilic groups, such as hydroxyl and carboxyl, and the reactive group promotes flocculant adsorption bridging between the molecules and aerosols because of the neutralization and chemical reaction [15,16].

The discovery and application of microbial flocculants has a history of more than 100 years. In 1876, Louis Pasteur discovered microbial flocculation while studying yeast. This is the first report on the phenomenon of microbial flocculants. In 1899, Bordet discovered that certain bacteria could also produce flocculation. The earliest bacterial flocculant-producing bacteria was Zoogloea, which was isolated from the activated sludge screened by Butterfield in 1935 [18]. In 1976, Nakamura et al. conducted systemic experiments on 214 species of different strains, and finally filtered 19 kinds of microorganisms with flocculation ability [18,19]. Thus, the study of microbial flocculants opened the curtain. More and more microorganisms are found to have or be capable of producing microbial flocculants. In addition, the bacterial species, such as *Paenibacillus* sp., *Alcaligenes latus*, *Pseudomonas aeruginosa*, *Bacillus* sp., *Rhodococcus* sp. and *Acinetobacter* sp, are innocuous in nature to the ecosystem when in the use of a bioflocculant [20,21]. Until now, it has been found that the microbial flocculants are mainly distributed in mold, bacteria, actinomycetes, and yeast. Common microorganisms that can obtain microbial flocculants include gram positive bacteria (*Rhodococcus erythropolis*, *Nocardia calcarea*, *Stink bugs bug nocardia*, *Corynebacterium*, etc.), gram negative bacteria (*Alcaligenes latus*, etc.), and other microorganisms (*Agrobacterium*, *Dematium*, *Pseudo single cell fungus*, *Eritrea’s bacillus genus*, *acinetobacter*, *Soy sauce aspergillus*, *Paecilomyces*, etc.) [7,9]. Among them, the flocculant produced by *Paecilomycin* has good flocculation effect on food wastewater, coal slurry wastewater, and textile wastewater [22], and the flocculant produced by *Rhodococcus erythropolis* [23] has good flocculation effect on fine coal wastewater, papermaking wastewater, and activated sludge.

Up until now, the application of bioflocculants in wastewater treatment have received more and more concerns. This article introduces the development status of microbial flocculation, the mechanism and application of the mechanism of flocculant, predicts the development trend, and gives suggestions on the research and application of flocculants.

## 2. Preparation of Microbial Flocculants

The formation of microbial flocculant is mainly related to the metabolic behavior of the microorganism itself. A large number of experiments show that the maximum flocculating activity appeared in the mid-late microbial metabolic behavior or stationary phase. This is due to the microbial metabolic activity or self-dissolution behavior, which leads to the flocculation activity that occurs in the middle or late stage of microbial metabolism behavior. The factors that affect the production of microbial flocculant include carbon source, nitrogen source, carbon and nitrogen ratio, temperature, pH, dissolved oxygen, culture time, and so on [24,25]. Some microorganisms need the stimulation of metal ions to produce the required biological flocculants [26,27].

### 2.1. Effect of pH Value on Production of Microbial Flocculants

Due to electrical neutralization, the surface charge and state of charge of flocculant molecules and suspended particles in water can directly affect the flocculation effect. The pH of the flocculation system will affect the surface charge and the charged state of the colloid particles, thus affecting the flocculation effect [28,29]. Different microbial flocculants have different sensitivity to pH value [30,31]. Microbial flocculant can only show the flocculation activity in a certain range of pH value [32,33]. When the change of pH of the reaction system exceeds this range, it will not only change the state of the charged state and the ability to neutralize the charge, but also change the surface properties of the particles [34]. The research showed that the flocculation of different substances by the same kind of microbial flocculants had different optimum pH value.

### 2.2. Effect of Temperature on Production of Microbial Flocculants

The effect of temperature on microbial flocculants is divided into indirect effects and direct effects. The indirect effect means that the microorganism has the most suitable growth temperature and the temperature at which the flocculant is generated, and the temperature affects the yield and performance of the flocculants by affecting the microorganism [35]. The direct effect is that the temperature directly affects the molecular motion of the microbial flocculant. Flocculation reaction has a better performance with the rise of the temperature, as it accelerates the speed of flocculants’ molecular movement. However, for protein flocculants when the temperature is too high, it leads to the degeneration of proteins and the destruction of space structure, which finally affects the performance of flocculation reaction. For some flocculants, which are composed of polysaccharide, the change of temperature has little influence on flocculation reaction; after high temperature treatment, the structure of polysaccharide flocculants has little change, and still has the ability to adsorb suspended solid. Therefore, the activity of flocculants is influenced by temperature and it is related to the flocculants’ character.

### 2.3. Effect of the Dosage of Microbial Flocculants

The dosage of flocculants is one of the factors that affects the performance of the reaction system [36]. Research shows the best dosage of microbial flocculants makes the adsorption of solid particles surface achieve saturation. At this time, adsorption quantity and adsorption bridging action most likely happen. Meanwhile, when the dosage of flocculants is out of range, the performance of flocculation would be influenced [37,38]. When the dosage is not enough, flocculating agents cannot flocculate ions in water completely; when adding too large a quantity of flocculants, reactions between flocculants’ molecules may occur, as a result, affecting flocculation performance [39]. Due to the co-working of adsorption bridging, network capturing, and other functions, even using the same kind of flocculants to treat different substances, the suitable dosage is different.

### 2.4. Effect of Type and Concentration of Metal Ions

In the application of flocculating agents, coagulants are always used to enhance flocculants’ performance. Different kinds of microbial flocculants used different metal ions as coagulant aids (i.e., Ca^2+^, Mg^2+^, Mn^2+^, Al^3+^) [40]. The presence of high concentrations of Ca^2+^ protects microbial flocculants from degradation enzymes. A suitable amount of metal ions has the ability to promote flocculation reaction, among which Ca^2+^ and Mg^2+^ ions have obvious effects [41,42], as they can promote the adsorption bridging between flocculant molecules and colloidal particles, neutralize a part of negative charge, and decrease the repulsive interaction between flocculant molecules and colloidal particles [43]. However, when adding too much metal ions, the activity of flocculants would be influenced, because redundant metal ions occupy an activated position of flocculant molecules and hinder adsorption between colloidal particles and flocculants’ surface.

## 3. Performances of Microbial Flocculants in Treatment of Various Wastewater

For the research of microbial flocculants, the most fundamental purpose is to be used in actual production and life. The sources of different wastewaters are different and the composition varies widely. The biological flocculants required to treat these wastewaters are also very different. There are many types of microbial flocculants, the flocculation mechanisms are different when treating different wastewaters, and the performances they exhibit are also different. Table 2 shows the microbial flocculants used in several common wastewaters. The specific components of these microbial flocculants in wastewater have a good flocculation effect.

### 3.1. Coal Slurry

Coal slurry is industrial wastewater which is in the process of wet-processing in the coal preparation plant [73,74]. The coal slurry wastewater is mainly a mixture of water and fine coal powder, and the solid particles are fine and the turbidity is high. It is influenced by Brownian motion, gravity, and the interaction between solid particle interfaces. Coal slurry wastewater has both the properties of a suspension and the nature of a colloid. Therefore, it is difficult to clarify naturally. Now, factories still use the high polymer flocculant to deal with coal slurry wastewater in general. However, the use of high polymer flocculant is not only expensive, but also can produce secondary pollution [75,76]. It is very important to find a kind of economic and safe flocculant to deal with coal slime water. In the early 1960s, R.W Smith used mycobacterium to deal with coal slurry and achieved good results [77]. Now, using microbial flocculant to deal with coal wastewater is not only efficient, fast, has no pollution and microbial flocculant, but also can degrade organic pollutants [78]. Compared with foreign countries, the research on the flocculation of coal slurry of microbial flocculant is earlier than China. Liu et al. [79] first studied the breeding of microorganisms for the production of coal slurry flocculant in China. Fine coal is hydrophobic and bacteria are hydrophilic. The surface of bacteria has a strong electron donor property, which interacts with fine coal, which can promote flocculation of slime wastewater [80,81]. In the experiment, the original *Phanerochaete chrysosporium* domestication and breeding of *Phanerochaete chrysosporium* was used as flocculant-producing bacteria. The experiment showed that the original *Phanerochaete chrysosporium* under the experimental conditions the maximum flocculation rate was 93.5%, flocculation acclimation *Phanerochaete chrysosporium* rate can reach the maximum of 88.68%, and UV mutagenesis and chemical mutagenesis ball *Phanerochaete chrysosporium*’s highest flocculating rate reached 95.08% and 95.04%, respectively [32,82]. The study also showed that the domestication breeding was helpful to improve the stability of the flocculation coal slurry [83,84,85].

### 3.2. Food Industry Wastewater

The food sector is one of the sectors that produces the largest amount of wastewater in industrial production, and it is also one of the sectors with the most complex wastewater components. Food wastewater includes grains, fruits and vegetables, meat, eggs, milk, poultry, and so on. The raw materials are wide and varied, and the water quality varies greatly, including starch, protein, oil, and so on, which are not easily melted, and the acid–base salt and sugar. Wastewater that has not been thoroughly processed during food or grocery processing has a high pollution load. Wastewater produced during the production of protein foods has a high nutrient content, which can easily cause eutrophication of water bodies, with dairy products being the most obvious [7,86]. This type of food wastewater is mainly organic pollutants. In 1974, the State promulgated the Water (Prevention and Control of Pollution) Law and amendments, which set limits and requirements for wastewater treatment and rational use of water resources [7,86].

The wastewater from the food processing industry contains a lot of organic matter and suspended matter. The wastewater discharged from the production enterprises, such as beer and gourmet powder, has high BOD and COD. If these wastewaters directly discharged into the environment, it will consume a large amount of dissolved oxygen in the water, directly leading to environmental deterioration. If treated with organic and inorganic flocculant, the flocculation recovery is not easy to be degraded. Experiments proved that the microbial flocculant has a better treatment effect on the food wastewater, and with flocculation recycling being biodegradable, no second pollution was formed.

### 3.3. Heavy Metal Wastewater Treatment

Heavy metal refers to a metal that has an atomic density of 4.5 g/mL or more. Heavy metal wastewater refers to wastewater containing nickel, cadmium, mercury, lead, chromium, copper, etc., produced in industrial production such as smelting, electronics, and chemical industries. Electroplating, mining, and chemical industries produce large amounts of heavy metal wastewater every year [87]. The discharge of these heavy metal wastewaters into the environment is extremely harmful to the environment and human health. For example, lead can directly damage brain cells, cadmium can destroy bone calcium, mercury can damage the central nervous system, and chromium can cause numbness and mental abnormalities in the limbs. Heavy metals not only have direct hazards, but many heavy metals can cause accumulation poisoning. Japan’s painful and watery disease episodes that shocked the world in the 1960s were caused by cadmium and mercury pollution. A mercury content of more than 0.1 mL per liter of drinking water can cause intense poisoning. However, even if the content is relatively low, long-term drinking will accumulate in the body, causing poisoning. The question of how to remove heavy metal ions in wastewater effectively and at the same time realize the wastewater reuse and recycling of heavy metals is the development direction of heavy metal wastewater treatment [88,89]. Microbial flocculant, as a green flocculant, is also applied to the process of flocculation of heavy metal wastewater [62,90,91,92]. The flocculating effect of microbial flocculants on metal wastewater is not only affected by the nature of microorganisms, but also by the affinity of microorganisms to metals. For example, bacillus flocculant has a good treatment effect on Cu^2+^ and Pb^2+^. The removal rate of Cd^2+^ by Pseudomonas can reach 93.5% [68]. The results show that the biosorption ability of the exopolysaccharides is much higher than that of the extracellular proteins in the extracellular polymers.

### 3.4. Papermaking Wastewater Treatment

Generally, there are three kinds of raw materials for papermaking: wood, non-wood, and recycled fiber, which are completed through 8 processes and 31 technologies [93,94]. In the process of production, 60 m^3^ fresh water is consumed per ton of paper, so the paper mill also consumes water [27,95]. If such wastewater cannot be effectively solved, it will easily cause harm to the aquatic environment [96,97]. In the study of papermaking wastewater treatment with the extraction of microbial flocculant, the flocculation effect is good and the removal rate of ammonia nitrogen can be as high as 96% under the optimum flocculation conditions. Fungi are widely used in pulp and paper mill wastewaters [26,98]. This is because compared with bacteria, fungi are more resistant to interference and have the ability to produce extracellular enzymes [99,100], the most common of which is white rot fungi. *Trametes pubescens* [101] and *Phanerochaete chrysosporium* [94] are two types of white rot fungi that have been studied intensively. They are capable of degrading lignin and phenolic compounds through the production of peroxidases and laccases [102,103]. Malaviya and Rathore [104] used *Merulius aureus*, an unidentified genus and *Fusarium sambucinum* to form a solidifying fungal complex for experimental research on the treatment of kraft paper wastewater. The first two bacteria belong to *Basidiomycetous fungi*, and the last one belongs to *Deuteromycetous fungus*. The raw materials for kraft paper production are bagasse and eucalyptus wood, and the resulting pulp color and COD are very high. The experimental results show that using this fungal complex can reduce the wood color and lignin content by 78.6% and 79.0%, respectively, and the COD removal rate is as high as 89.4%. In addition, *Pleurotus sajor caju* and *Rhizopus oryzae* species were studied in the experiment. The former belongs to white rot fungi and the latter belongs to soft rot fungi. By using this fungal complex to treat the wastewater produced by the secondary treatment of bleached sulfate blue eucalyptus, the color and COD of the wastewater were significantly reduced. After 10 days of incubation, the removal rate of COD was 74–81% [103]. Liu et al. [105] selected a mechanical pulp for laboratory test. The main component of this mechanical pulp is *Aspergillus* niger poplar alkaline peroxide. Orthogonal experiments optimized reaction conditions. The results showed that when the inoculation amount was 3% and the reaction pH was 6, the continuous shaking at 160 rpm for 60–72 h, and the reaction was maintained at a temperature of 30 °C, the effect was the best. At this time, the COD removal rate was 60%, and the turbidity and color removal rates were 77% and 43%, respectively. Experiments can obtain about 97% of methyl tert-butyl ether (MTBE) extract [105].

### 3.5. Textile Wastewater Treatment

The textile industry is one of the main producers of industrial wastewater. Different resources and raw materials consume different amounts of water. For example, the pre-treatment water consumption of cotton and wool processing is very large. In contrast, the water consumption of synthetic fibers is much smaller. The amount of water required by different processes also varies. Generally speaking, wet processing operations consume more water. The composition of textile wastewater is also complex. In addition to basic acids and alkalis, dyes that meet various color needs, there are differences caused by different raw materials, such as cotton waste from cotton textile wastewater, proteins and oils from wool textile wastewater, and fiber debris from fiber textile wastewater. In addition, in order to improve the quality of textiles, many additives are added to the textile processing process, which may directly or indirectly cause excessive levels of organic pollutants in textile wastewater and serious excessive levels of heavy metal ions [106]. Therefore, if we only look at the global impact from the perspective of the impact on the environment, it is estimated that the textile industry is the industry with the highest water demand. According to World Bank estimates, textile wastewater accounts for about 17% to 20% of total industrial wastewater. A typical medium-sized textile mill consumes about 200 L/d/kg of textiles. What is more serious is that textile wastewater with a high pollution load can easily cause secondary pollution [107]. The biological treatment of textile wastewater is generally based on the treatment of dissolved matter in wastewater [70]. The removal efficiency of the microbial method is related to the load of the microorganism itself, and is also affected by the oxygen concentration, temperature, and organic load/dye ratio in the system [106,108,109]. Microbial domestication is a good method to obtain microorganisms adapted to the targeted environment. Microorganisms gradually adapt to textile dyes. Newly grown strains are more adapted to environmental requirements. Whether the strains grow or secrete enzymes that solve difficult-to-degrade dyes, such as laccase, tyrosinase, and aminopyrine *N*-demethylase, this can reduce the harm of textile wastewater to the environment [110]. The efficiency of bioflocculation and biodegradation depends on the adaptability of the microorganisms themselves and the activity of the enzymes produced. In recent years, through the domestication of microorganisms, a large number of microorganisms adapted to the targeted environment have been isolated, thereby obtaining a large number of enzymes. Bioflocculation and biodegradation treatment of wastewater can reduce water consumption and produce no harmful substances in metabolism. Biological treatment is more ecologically friendly, low cost, and competitive in the industry [109].

In the research of treating textile wastewater, the separation of effective microorganisms and the extraction of related enzymes are very interesting research aspects in biology. So far, in the process of treating textile wastewater, a variety of microorganisms (including bacteria, fungi, and even algae) with flocculation and degradation ability have been obtained. In comparison, the bacterial response is more sensitive and the fungus is more adaptable. Algae has a good treatment effect on metal ions and special biological macromolecules. Fungal cultures are able to adapt their metabolism to changing environmental conditions. For them, it is a capability related to survival. Here, intracellular and extracellular enzymes contribute to metabolic activity. These enzymes are capable of degrading various dyes in textile wastewater. Due to the presence of these enzymes, fungal cultures appear to be suitable for degrading dyes in textile wastewater. These enzymes are *Lignin peroxidase* (LiP), *Laccase* and *Manganese peroxidase* (MnP) [111], white rot fungus *Pleurotus eryngii* [112], and *Penicilliumsim plicissimum* [113]. However, white rot fungi *coriolopsis* sp. [111] have long growing seasons, high environmental requirements, and unreliable enzyme production. The main problem with the use of fungi alone is that the system is unstable. It takes 20–30 days for the bacteria to start growing and the fungi will no longer dominate the system and degrade the dye [114]. It has almost no flocculation degradation.

Due to its unique ability to remove azo dyes, algae have received more attention and have been used more widely in the removal of dyes in textile wastewater. In addition, the bio-adsorption process that uses algae waste for decolorization is a practical alternative to expensive activated carbon and other materials.

Generally speaking, bacteria can also degrade organic pollutants in textile wastewater. Bacteria mainly degrade azo dyes. This is because the azo reductase produced by bacteria can break the azo bond under anaerobic conditions, thereby destroying the dye structure and facilitating the further processing of intermediates. In addition, the degradation of disperse red 78 by bacteria (Pseudomonas) was 37% higher than that by the fungal system (Aspergillus oryzae NCIM-1146) [108]. This article reports the degradation pathways and pharmacodynamics of degradation products. Holkar et al. reported that under natural conditions, anthraquinone-based active blue 19 degraded 90% in one day and night, while Wang et al. reported that under anaerobic conditions, anthraquinone-based active black 5 was degraded in five days and nights, at 92% [115,116].

### 3.6. Printing and Dyeing Wastewater Treatment

Printing and dyeing wastewater refers to the wastewater generated during the printing and dyeing processing of cotton, wool, hemp, fiber, and their mixed textiles. The printing and dyeing wastewater contains textile impurities, dyes, and additives. Printing and dyeing wastewater is mainly produced in several processes such as desizing, scouring, bleaching, mercerizing, whitening, dyeing, and printing. The printing and dyeing wastewater contains high concentrations of textile impurities, dyes, auxiliaries, oils, and slurries [117,118]. Although the pollutants in different printing and dyeing products and the wastewater discharged from different printing and dyeing processes vary greatly, overall, the high content of organic pollutants, large alkalinity, unstable water quality, high chemical oxygen demand, poor biodegradability, and low BOD/COD ratio are the main characteristics of printing and dyeing wastewater [119,120,121]. The chroma of printing and dyeing wastewater is higher than other general wastewater [122,123], and the composition is more complicated than general wastewater. There are many varieties of dyes and intermediates, and even toxic and harmful substances, such as hexavalent chromium and aniline dyes [124,125].

## 4. Mechanisms of Bioflocculation

In terms of chemical composition, it is mainly produced by microbial metabolism with a flocculation activity of protein, polysaccharide, and some of the flocculant also contains inorganic metal ions spatial structure from the point of view, there are two kinds of microbial flocculants micro stereoscopic structure: fibrous structure and ball. Microbial flocculants have been investigated in different kinds of wastewater treatment as well. Pant and Adholeya [126] used bacterial, fungal, algal, and plant-based systems in the color removal of distillery effluent. In flocculant preparation, different kinds of microbial species have been studied and reported, such as *Enterobacter* sp. [13,116], *Halomonas* sp. [127,128], *Citrobacter* sp. [129], *Bacillus* sp. [16,130,131,132,133,134,135], and *Rhodococcus erythropolis* [136,137,138].

In general, development of flocs formed mainly has several steps: (a) Flocculant molecules are gradually dispersed in the solution; (b) Collisions with other particles in the particle carrying a flocculant adsorption; (c) Adsorption of flocculant molecules on the surface of particles [139,140]; (d) Small aggregates grow larger and stronger through continuous collision and adsorption [126]. There have been many hypotheses about the mechanism of microbial flocculant, such as Butterfield’s hypothesis about adhesion, Grabtree’s Ester composite hypothesis based on the study about PHD (Poly-β-hydroxybutyric acid), and Friedman’s extracellular fiber theory [12,141]. However, generally speaking, the hypotheses accepted at the moment are adsorption bridging mechanism, charge neutralization mechanism, chemical reaction mechanism, mechanism of volume sweeping effect, etc. The particle adsorption on the surface of the flocculant is the result of the combined effect of these mechanisms. It is a complicated process as when processing wastewater by microbial flocculant, the flocculation mechanism of many microbial flocculants is not only a single one, but the result of several mechanisms working together [142]. Moreover, when the effect of microbial flocculant was changed, the mechanism was not identical.

### 4.1. Adsorption Bridging

Flocculation process refers to the process of polymerization of multiple particles [119,143]. In the process of flocculation, there are colloid particles from a stable state [144,145]. According to adsorption bridging theory, microbial flocculant is a kind of chain macromolecule which has the characteristics of high molecular weight and low charge density [119,143]. There are adsorbed active sites on the molecule. Colloidal particles are adsorbed on the molecule by the adsorption of the active site [71,146]. At the same time, multiple microbial flocculant large chainlike molecules through hydrogen bond intermolecular van der Waals forces the particle bypass to be formed by a three-dimensional network structure and set down [147]. The length of the flocculant should be long enough to extend from the surface of the particle to the other in order to effectively build bridges [148]. The space of the flocculation should be large enough, that is, the adsorbing capacity should be large enough, in order to adsorb the attachment particles in the other segment [129]. The suitable chain length and the adsorption capacity can make the bridge contact more resistant to damage and improve flocculation efficiency.

### 4.2. Charge Neutrality

The flocculant and the adsorption site are opposite charges in many practical cases [149]. There is a negative correlation between chemical oxygen demand and conductivity [150]. The colloid in the water generally carries a negative charge. When adding microbial flocculant, a neutralizing effect occurs between them as the flocculant is macromolecules with a positive charge on the surface [143]. Due to the reduction of the surface charge of the particles, that is, the Zeta potential is reduced, the repulsive force between the particles is reduced [151,152]. By the attraction of van der Waals, colloid and suspended solids gathered to form flocs, then the colloid destabilization, after this, colloids and colloid, or colloid and microbial flocculant were flocculated into large particles [153,154]. The flocculation effect can be influenced by the pH value of the flocculation reaction system due to the change of the water colloid or flocculant [155,156,157,158]. Similar to that, many studies have found that when Zeta potential is close to zero, the flocculation effect is best. Thus, when there is a charge reversal, the attraction between the particles may turn to repulsion, and the particles may be dispersed again.

### 4.3. Chemical Reactions

Microbial flocculant belongs to the biological macromolecules. Some of the groups on the molecule react chemically with some of the groups on the surface of the colloid. Then flocculation into larger particles precipitate out of the water. The modification of microbial flocculant (adding or reducing the reactive groups on the flocculant molecules) can also change the flocculation effect of the reaction system. The temperature can affect the flocculation effect because the temperature can directly change the activity of the reaction group.

### 4.4. Volume Sweeping

When added to a certain amount of microbial flocculant, flocculation floc increases with the process continuously, and, settling down, relies on gravity. In the process of sedimentation, floc, just like a sieve, can quickly sweep the volume and capture some colloidal particles dispersed in water material, then become a larger floc. Thus, the colloidal material can be separated from the water by mechanical precipitation. In the process of forming a sieve, the network needs enough nodes to form a sieve. The concentration of impurities in water is inversely proportional to the dosage of microbial flocculant. The higher the concentration of impurities, the less the amount of microbial flocculant. On the contrary, the less impurities in the water, the more flocculant would be needed to achieve the effect of volume sweep.

## 5. Prospects of Microbial Flocculants

In recent years, microbial flocculant has been identified as a potential solution to a large number of wastewater treatment. However, in practical applications, microbial flocculants work very limited in engineering scale, mainly concentrated in the experimental stage. Some problems, such as a wide variety of microorganisms, microbial variability, and so on, are still widely used in microbial flocculant-caused obstacles. In addition, the screening of efficient flocculants produces bacteria and the high cost of microbial flocculant produces bacteria. The author thinks that the microbial flocculant can be further studied in several aspects.

### 5.1. Applications of Cheap Culture Media

Carbon source and nitrogen source are the main sources of the growth of microorganisms [159,160]. The use of yeast extract, glucose, and so on in the process of laboratory culture is too large and the cost is too high, which is not conducive to mass production and application. Wastewater, food wastewater, and other wastewater are complex components, which can provide energy for microorganisms while reducing the content of organic matter and nitrogen in wastewater [57,161], given the difference of the contents of the different wastewaters. The reasonable ratio of different wastewater can effectively reduce the external energy, and in the process of building a cheap medium, the water purification effect can be better [162]. Moreover, while screening suitable media, the culture conditions of the microbial flocculant strain can be continuously optimized [163,164], so as to obtain a microbial flocculant with lower cost and a better flocculation effect.

### 5.2. Production of Composite Bioflocculants

A single biological flocculant is usually selective. When the composition of wastewater is complex in actual production and life, the application of biological flocculants is limited. It is found that in the process of screening bacteria, the mixed bacteria are sometimes more effective than single species [145,165]. Therefore, screening the production bacteria of the composite flocculant in the application process [166] and exploring the synergistic effect of different strains in producing flocculant in the wastewater treatment is of great significance for culturing the composite flocculant bacteria. 

### 5.3. Understanding of Bioflocculation Mechanisms

Pay attention to the specificity and general characteristics of microbial flocculation in wastewater treatment in order to make the microbial flocculant more rapid and effective in the treatment of different wastewater. In addition, some polysaccharide microbial flocculants have special effects of anti-inflammatory, anti-oxidation, and anti-oxidation [167]. Moreover, some polysaccharide-based flocculants can be used as reducing agents and stabilizers to promote the synthesis and separation of some experimental drugs [168].

By studying the effect of similar cells, there is potential to explore effective gene fragments, and through the research of gene transposon, explore the function of training high flocculation effect of flocculants producing bacteria and simultaneous desulfurization and denitrification removal or other organic matter producing bacteria, and explore the application of flocculant-producing bacteria in special water bodies. 

Due to the shortage of energy and the serious environmental pollution caused by excessive use of fossil fuels, biofuels have been considered as a potential substitute in recent years. Among them, biodiesel is one of the most commonly used biofuels. Moreover, among many potential biodiesel raw materials, microalgae have the advantages of large output and fast accumulation [169]. Microalgal bacterial flocs is a popular and promising microalgae collection and wastewater treatment method [144,170,171]. Lei et al. [172] have reported that microbial flocculant flocculation-flotation technology can effectively harvest chlorella, and the use of composite flocculants can also enhance the harvest of chlorella [173]. This study provided some insights on the formation of flocs and provided a theoretical basis for further improving the recovery of chlorella and removing nutrients from seafood wastewater [174,175,176].

## 6. Conclusions

In this review, the principle of flocculation, the synthetic conditions and influencing factors of microbial flocculants, and the application of flocculants in production and life are introduced. It is helpful to understand the mechanism and development status of microbial flocculants, such as the present situation of microbial flocculants, the flocculation mechanism of microbial flocculants, the synthetic conditions and influencing factors of microbial flocculants, and the application of microbial flocculants in wastewater treatment. The performance of microbial flocculants has focused on the application of microbial flocculants in coal slurry, food industry, heavy metal, papermaking, textile, printing, and dyeing wastewater. The development prospects of microbial flocculants are described in this paper. 

## Figures and Tables

**Table 1:** Advantages and limitations of three types of flocculants.

Type	Main Ingredients	Advantages	Disadvantages	References
Inorganic flocculants	Aluminum salt polymers and iron salt polymers	Low-cost Easy to get	High pollution Difficult to degrade	[7,9]
Organic polymer flocculants	Polyacrylamide and their derivatives	Less dosage Fast flocculation Easy to separate	Difficult to degrade Easy to cause two pollution	[12,14]
Microbial flocculants	Microbes and their metabolites	Effective Low-cost Security extensive source	Slow effect Environmental susceptibility	[15,16]

**Table 2:** Microbial flocculants used in several common wastewaters.

Wastewater Type	Contaminant Category	Flocculating Microorganisms	References
Coal slurry wastewater	Coal waste slurry	*Azotobacter chroococcum*	[44]
	Coal slurry	*Rhodopseudomonas spheroides*	[45]
	Coal-water slurry	*Nitrogen-fixing bacteria* *Mycobacterium tuberculosis* *Escherichia coli White rot fungi Yeast Aspergillus niger*	[46]
	Fine coal	*Bacillus subtilis*	[47]
Food wastewater	Bioethanol mill wastewater	*White-rot fungus Trametes versicolor* INACC F200	[48]
	Suspended solids Metal ions	*Bacillus subtilis* R 23 *B. lichenifomis* ATCC 9945A *B. lichenifomis* CCRC 12826 *B. licheniformis* CICC10099	[49]
	Poultry slaughterhouse wastewater	*Comamonas sp.*	[50]
	Starch wastewater	*Bacillus mucilaginosus* MBFA9	[51]
	Starch wastewater	*Bacillus licheniformis* X14	[52]
	dairy wastewater Fe Al Mn Zn COD	*Terrabacter sp.*	[53]
Municipal wastewater	Harmful algae	*Klebsiella pneumoniae strain* NY1	[54]
Landfill leachates	Humic acids	*Rhizomonas sp.*	[29]
Heavy metal wastewater	Cu(II)	*Ultrasonic assisted Spirulina platensis*	[55]
	Cu(II)	*Sulphuric acid modified Spirulina platensis*	[56]
	Pb(II)	*Paenibacillus sp. strain* A9	[57]
	Pb(II)	*P. polymyxa* CCTCC M206017	[58]
	Pb(II) Zn(II)	*Paenibacillus sp. strain* A9 (MBFA9)	[59]
	Ni(II)	*Paenibacillus polymyxa* GA1	[60]
	Cr(II) Ni(II)	*Herbaspirillium sp.* CH7 *Paenibacillus sp.* CH11 *Bacillus sp.* CH15 *Halomonas sp.*	[61]
	Cu, Zn, Pb and Cd	*Paenibacillus polymyxa*	[62]
	Fe (III) Cd(II)	*Aspergillus niger*	[63]
	Minerals	*Rhodococcus opacus*	[64]
Papermaking wastewater	COD_Cr_ content absorbance	*Aspergillus niger*	[65]
	Detergents cellulose	*Bacillus subtilis* *Rhodococcus* *Bacteroides succinicum* *White rot fungus bacterial*	[66]
	COD Colority	PSBF	[67]
Textile wastewater	Cu(II), Pb(II) and Cd(II)	*Bacillus sp.* *Pseudomonas sp.*	[68]
	Biavin medium blue dye Cr(II) Ni(II)	*Herbaspirillium sp.* CH7 *Paenibacillus sp.* CH11 *Bacillus sp.* CH15 *Halomonas sp.*	[61]
	Anthraquinone based Reactive Blue 19	*Enterobacter sp.* F NCIM 5545	[69]
	Acid and reactive dyes	*Pleurotus osstreatus* *Aspergillus niger* *Penicillium spp.*	[70]
Printing and dyeing wastewater	Congo red (CR)	*Klebsiella peneumoniae*	[71]
	Methylene blue	*Paenibacillus polymyxa* GA1	[72]
